# Incremental value of contrast myocardial perfusion to detect intermediate versus severe coronary artery stenosis during stress-echocardiography

**DOI:** 10.1186/1476-7120-8-16

**Published:** 2010-05-06

**Authors:** Nicola Gaibazzi, Fausto Rigo, Angelo Squeri, Fabrizio Ugo, Claudio Reverberi

**Affiliations:** 1 Cardiology Division, Azienda Ospedaliero-Universitaria di Parma, Parma, Italy; 2 Cardiology Division, Umberto I° Hospital, Mestre-Venice, Italy

## Abstract

**Background:**

We aimed to compare the incremental value of contrast myocardial perfusion imaging (MPI) for the detection of intermediate versus severe coronary artery stenosis during dipyridamole-atropine echocardiography (DASE).

Wall motion (WM) assessment during stress-echocardiography demonstrates suboptimal sensitivity to detect coronary artery disease (CAD), particularly in patients with isolated intermediate (50%-70%) coronary stenosis.

**Methods:**

We performed DASE with MPI in 150 patients with a suspected chest pain syndrome who were given clinical indication to coronary angiography.

**Results and discussion:**

When CAD was defined as the presence of a ≥50% stenosis, the addition of MPI increased sensitivity (+30%) and decreased specificity (-14%), with a final increase in total diagnostic accuracy (+16%, p < 0.001). The addition of MPI data substantially increased the sensitivity to detect patients with isolated intermediate stenosis from 37% to 98% (p < 0.001); the incremental sensitivity was much lower in patients with severe stenosis, from 85% to 96% (p < 0.05), at the expense of a higher decrease in specificity and a final decrease in total diagnostic accuracy (-18%, p < 0.001).

**Conclusions:**

The addition of MPI on top of WM analysis during DASE increases the diagnostic sensitivity to detect obstructive CAD, whatever its definition (≥50% or > 70% stenosis), but it is mainly driven by the sensitivity increase in the intermediate group (50%-70% stenosis).

The total diagnostic accuracy increased only when defining CAD as ≥50% stenosis, since in patients with severe stenosis (> 70%) the decrease in specificity is not counterbalanced by the minor sensitivity increase.

## Background

Standard pharmacological stress-echocardiography (SE) has demonstrated suboptimal sensitivity to detect intermediate coronary stenosis, particularly in patients with single-vessel disease, not differently from other techniques based on the detection of inducible wall motion abnormalities [1-3]. Nonetheless the diagnosis of a coronary stenosis between 50% and 70% in diameter is relevant to the patient, since it may portend an unfavorable prognosis when associated with reduced coronary flow reserve or fractional flow reserve [2,4].

Both the quantitative measurement of myocardial blood flow reserve and the more practical visual assessment of myocardial perfusion (MPI) during contrast-SE have demonstrated incremental sensitivity on top of wall motion (WM) analysis to diagnose obstructive coronary artery disease (CAD) [5-10].

The study hypothesis is that the potential incremental diagnostic sensitivity and accuracy of MPI during dipyridamole-atropine echocardiography (DASE) is driven by the higher sensitivity in the subset of patients with isolated intermediate coronary stenosis. We performed DASE with the addition of MPI in 150 consecutive patients with a chest pain syndrome who were given clinical indication to elective coronary angiography and we analyzed the diagnostic results based on the presence and grade of coronary artery stenosis.

## Methods

### Patients

All patients who were clinically indicated elective coronary angiography between March 2008 and January 2009 for a recent chest pain syndrome were asked their informed consent to be enrolled in the study; the ones who consented underwent DASE within 2 weeks before coronary angiography, in the absence of the following exclusion criteria:

#### Exclusion criteria

a) left ventricular ejection fraction (LVEF) < 30%, b) severe valvular disease, c) sustained ventricular arrhythmias or hemodynamic instability, d) insufficient acoustic windows, e) known allergy to sulfonamides.

The study complied with the Declaration of Helsinki. All patients gave written informed consent to the study protocol, which was approved by the Institutional Review Board of our Hospital.

### Dipyridamole-atropine echocardiography

**Figure 1:**
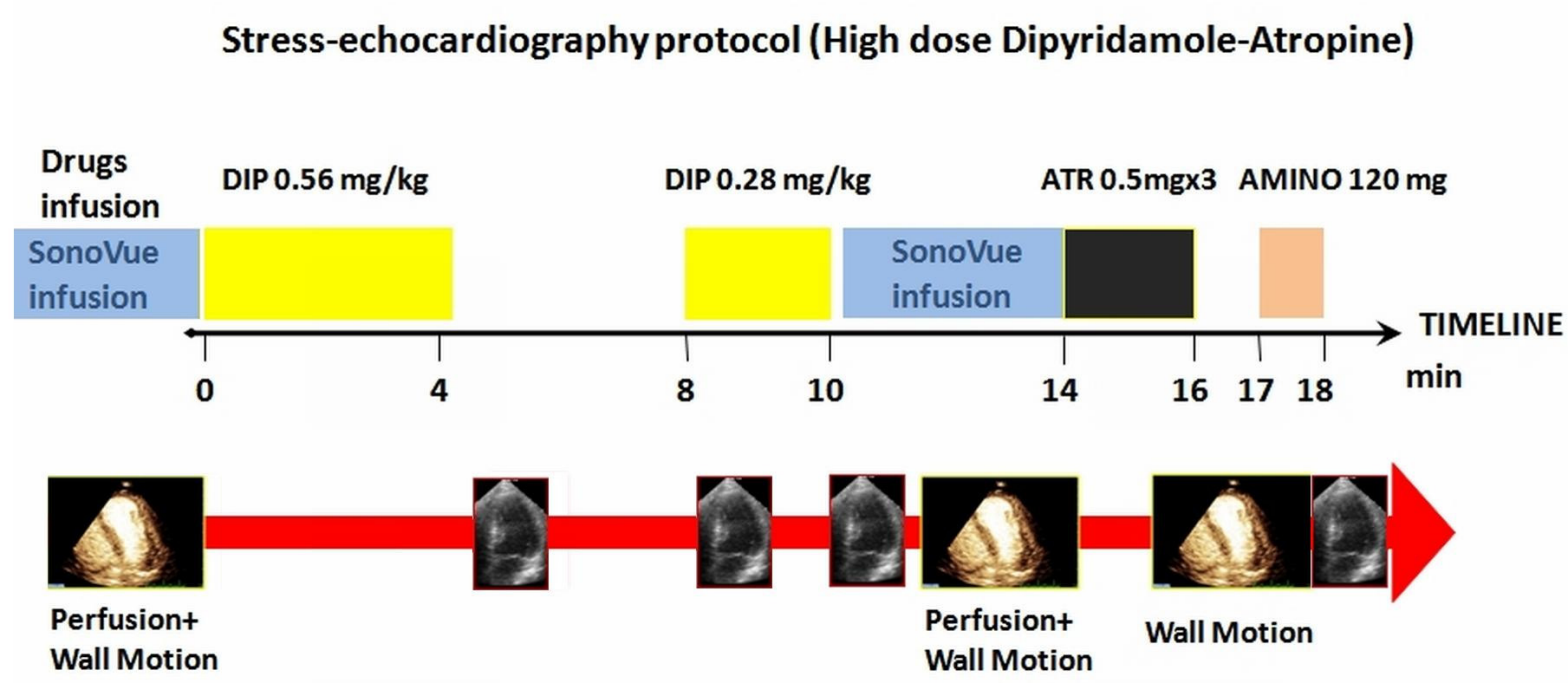
**Stress echocardiography protocol**. Abbreviations as in table 1.

The contrast stress protocol is shown in Figure 1. All images were acquired in the apical 4, 2 and 3 chamber views using S5 broadband transducer (Philips, Eindhoven, Netherlands). Wall motion and myocardial perfusion were assessed at rest and at peak stress. Sonovue (Bracco, Milan, Italy) was infused using a rotating pump (BR-NF100, Bracco, Geneva, Switzerland) at rest and 1 minute after completion of dipyridamole infusion. Low power (0.10) continuous imaging was performed for WM assessment. For MPI image acquisition a flash-replenishment sequence (destructive pulse of 8 frames at a mechanical index of 1.0, followed by low power imaging for 10 cycles) was used both in the continuous (40 frames/sec) and triggered mode (end-systolic at every cardiac cycle) [5].

Care was taken to opacify the left ventricle and the myocardium homogeneously with minimization of attenuation so that it is confined to the atrium prior to acquisition of contrast imaging. To achieve this the infusion rate and gain settings are adjusted (e.g. if the contrast attenuation is seen in the LV cavity, infusion rate is reduced and if the attenuation is optimal, then gains are increased for optimal opacification). Gain settings are considered optimal if the destructive impulses result in complete clearance of microbubbles from the myocardium with very little cavity contrast destruction.

#### Interpretation of WM and MPI

Contrast regional WM analysis was evaluated at baseline and at peak stress by using a semi quantitative wall motion score (normal, hypokinesia and akinesia) on a 17-segment model of the left ventricle, according to the recommendations of the European Association of Echocardiography [11].

Abnormal WM was defined as the occurrence in at least one segment of either a new dyssynergy in a region with normal rest function or worsening of rest dyssynergy. Resting akinesia becoming dyskinesia or fixed WM abnormalities were not considered a criterion for positivity.

Myocardial perfusion was considered as normal if the myocardium was fully replenished 1.5-2 seconds after the end of flash impulse at peak hyperemia and as abnormal if the myocardium replenishment was delayed beyond 2 seconds. The cut-off for normal replenishment at rest was considered 4-5 seconds after the flash impulse. A perfusion defect was scored as fixed or reversible based on its presence or absence at rest. A reversible defect in ≥1 segment was defined as abnormal MPI.

Wall motion and MPI were analyzed separately at 2 different time points by consensus by 2 observers. Disagreement was resolved by a third observer.

### Quantitative Coronary Angiography

Quantitative coronary angiography (QCA) was performed by a cardiologist (F.U.), unaware of the echocardiography results. Any evident stenosis was measured (Qangio XA version 7.0, Medis, Leiden, the Netherlands) and expressed as percent narrowing using the nearest normal-appearing region as the reference. An intermediate stenosis was defined as luminal stenosis in one or more coronary arteries or major branches between 50% and 70%, while a severe stenosis was defined as > 70% narrowing.

Patients were divided into subgroups: a) normal coronaries or < 50% stenosis, b) stenosis between 50% and 70% as the worst coronary lesion (INTERMEDIATE group) or c) at least one > 70% stenosis (SEVERE group). Patients with left main disease ≥50% were also included in the SEVERE group. Multivessel disease was defined when more than one of the three major coronary arteries (or left main trunk) was affected by a ≥50% stenosis. Coronary territories were assigned according to guidelines [11].

### Statistical analysis

Continuous variables were presented as mean and standard deviation and were compared using the Student *t *test. Categorical variables were examined with a chi-square test when appropriate (expected frequency > 5), otherwise a Fisher exact test was used. Sensitivity, specificity and accuracy were calculated using standard definitions and were presented with 95% confidence intervals (CI). Differences between sensitivity, specificity and accuracy using WM or WM+MPI were analyzed using McNemar's test. A value of p < 0.05 (2 sided) was considered significant. Interobserver and intraobserver agreement was determined for both WM and MPI in 30 randomly selected patients. Results were presented as a percentage with a corresponding kappa value. Statistical analysis was performed with SPSS version 15.0 (SPSS Inc, Chicago, Ill).

## Results

### Clinical data

169 consecutive patients met the inclusion criteria; 11 denied informed consent and 8 met exclusion criteria (6 insufficiently echogenic and 2 reporting known allergy to sulfonamides). 150 patients were finally enrolled. Mean age was 70 ± 9, 78 (52%) were males, 115 (77%) had at least 2 traditional risk factors for CAD, 40 (27%) had diabetes mellitus and 51 (34%) had a previous myocardial infarction or percutaneous coronary intervention; 47 subjects (31%) had a final angiographic result of single vessel-disease, 55 (37%) multivessel, while 48 (32%) had normal or < 50% stenosed coronary arteries. All demographic and clinical data were comparable between the INTERMEDIATE and the SEVERE group.

### Stress echocardiography

The prevalence of reversible WM abnormalities was higher in the SEVERE group when compared with the INTERMEDIATE group (p < 0.001); in contrast, the prevalence of MPI abnormalities was not different between the two groups (Table 1).

**Table 1:** Clinical characteristics and echocardiographic data in the 3 angiographic categories, according to QCA results.

Study group n = 150	No CAD	CAD 50%-70%	CAD > 70%	p value
Patients n (%*)	48	41	61	-

Age, mean(± 1 SD), y	64 (10)	69 (9)	70 (9)	ns

Men, n (%)	26 (54)	24 (59)	40 (65)	ns

Risk factors ≥2, n (%)	37 (77)	34 (85)	51 (84)	ns

Hypertension, n (%)	32 (67)	30 (73)	42 (69)	ns

Diabetes Mellitus n (%)	6 (13)	12 (29)	19 (31)	ns

Prior myocardial infarction or PCI n (%)	11 (23)	13 (32)	23 (38)	ns

Presence of baseline WM abnormalities, n %	10 (21)	14 (34)	24 (39)	ns

Baseline LVEF, %	57 ± 8	54 ± 9	52 ± 10	ns

Abnormal WM, n (%)	8 (17)	15 (37)	52 (85)	p < 0.001

Abnormal myocardial perfusion n (%)	15 (31)	40 (98)	58 (95)	ns

Peak RPP mean(± 1 SD)	15124 (3140)	15416(3684)	14615(2705)	ns

Risk factors considered were: hypertension, diabetes, current smoking, hypercholesterolemia, familiar history of premature coronary artery disease (CAD) and obesity. The "No CAD" group includes patients with < 50% CAD. P value refers to difference between the 2 disease groups "CAD 50%-70%" and "CAD > 70%". QCA = quantitative coronary angiography, PCI = percutaneous coronary intervention, LVEF = left ventricular ejection fraction, WM = wall motion, MPI = myocardial perfusion imaging, RPP = rate-pressure product

**Figure 2:**
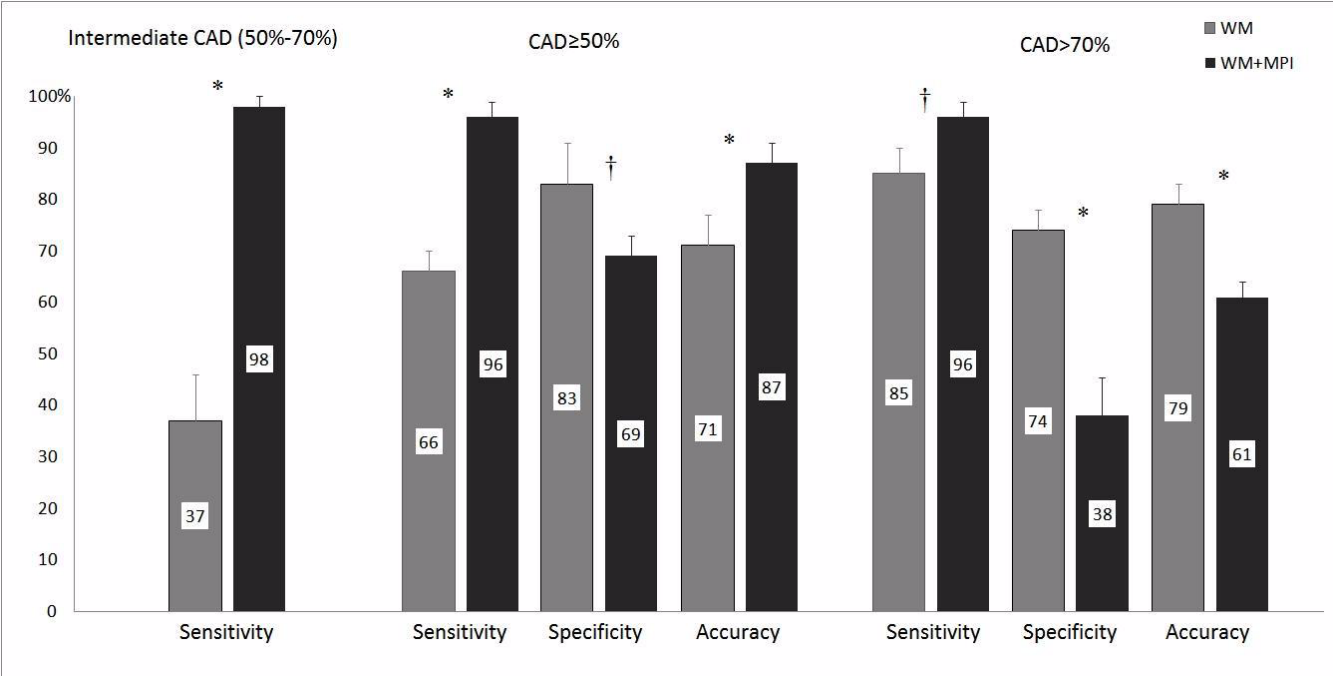
**Sensitivity, specificity and accuracy for each subgroup**. Accuracy data with corresponding 95% confidence intervals for wall motion and wall motion+myocardial perfusion imaging to detect patients with coronary artery stenosis between 50% and 70%, ≥ 50%, or > 70%. In the intermediate (50%-70% stenosis) group only sensitivity can be calculated, since in this case patients with a > 70% stenosis cannot be classified, although key to specificity and accuracy measurement. In the CAD > 70% an abnormal test with angiographic 50%-70% stenosis is considered a false positive. * p < 0.001, † p < 0.05 compared to wall motion criteria.

Sensitivity, specificity and accuracy for WM and WM+MPI in patients with any stenosis ≥ 50% (INTERMEDIATE+SEVERE groups) or > 70% only (SEVERE group) are reported in Figure 2, along with sensitivity data for the INTERMEDIATE group.

Sensitivity using WM+MPI resulted significantly higher than using WM data only, whatever the cut-off used to define CAD. The sensitivity increase was highest in the INTERMEDIATE group, from 37% (CI 25%-47%) to 98% (CI 89%-100%), (p < 0.001).

The sensitivity increase of WM+MPI for CAD ≥50%, from 66% (CI 61%-70%) to 96% (CI 92%-99%) (p < 0.001), was only partially counterbalanced by the specificity decrease from 83% (CI 70%-92%) to 69% (CI 61%-74%) (p < 0.05); MPI total accuracy resulted higher compared with standalone WM analysis, 87% (CI 82%-93) vs 71% (CI 65%-78%), (p < 0.001). On the contrary, in the SEVERE group the decrease in specificity obtained with the addition of MPI data was much higher, from 74% (CI 67%-79%) to 38% (CI 29%-49%, p < 0.001), than the sensitivity increase, from 85% (CI 76%-91%) to 96% (CI 92%-99%), p < 0.05), with final lower total accuracy, from 79% (CI 72%-83%) to 61% (54-65) (p < 0.001).

The addition of MPI identified 25 more true positive patients than WM in the INTERMEDIATE group; among them, in 13 patients (52%) the intermediate stenosis detected was in the anterior circulation (anterior descending coronary artery), in 10 patients (40%) in the posterior circulation (right or circumflex coronary artery) and in 2 patients (8%) both in the anterior and posterior circulation.

The addition of MPI identified 6 more true positive patients than WM in the SEVERE group, 5 with stenosis both in the anterior and posterior circulation (multivessel disease) and 1 with a single stenosis in the anterior circulation (single-vessel disease).

**Figure 3:**
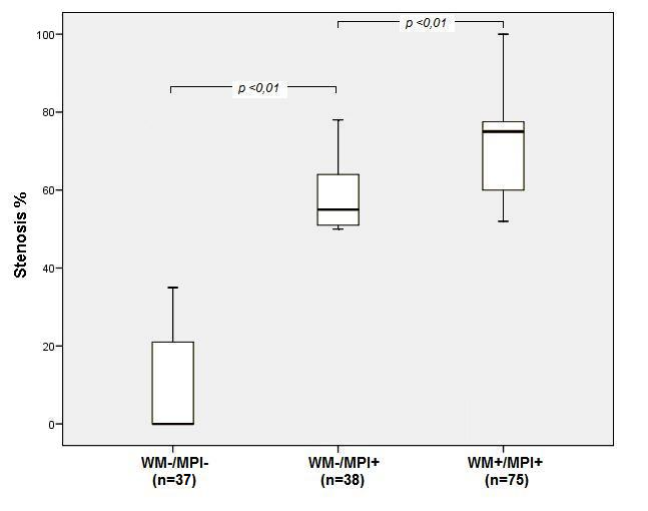
**Average stenosis diameter**. Average stenosis diameter in patients who underwent QCA, classified into three subgroups based on SE results: MPI-/WM-, MPI+/WM-, MPI+/WM+. Abbreviations as defined in table 1.

Figure 3 shows the average stenosis diameter in patients who underwent QCA, classified into three subgroups based on SE results: MPI-/WM-, MPI+/WM-, MPI+/WM+.

**Figure 4:**
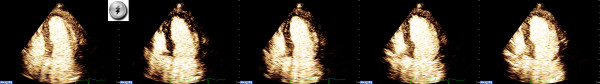
**Flash replenishment sequence**. Assessment of myocardial perfusion after dipyridamole. From left to right: uniform transmural perfusion seen in apical 4-chamber before flash, then images taken 1, 3, 4 and 8 cardiac cycles after microbubbles destruction. Perfusion defects become apparent after flashing (flash icon in the figure) both in the anterior descending and circumflex coronary arteries perfusion territories, still detectable after 8 cycles; the patient had no clear WM abnormality, even if mild tardokinesia of the septum was suspected. Angiography confirmed obstructive two-vessel disease with both stenosis ranging between 50% and 70%. Abbreviations as defined in table 1.

Figure 4 shows a typical DASE examination resulting positive for MPI after dipyridamole with normal peak WM, in a patient finally diagnosed with two-vessel 50%-70% coronary artery stenosis (see also Additional File 1).

### Intra/interobserver variability

The intraobserver agreement on the presence or absence of reversible visual MPI or WM abnormality after dipyridamole is respectively 97% (Kappa = 0.91) and 100% (Kappa = 1); the interobserver agreement data is respectively 93% (Kappa = 0.83) and 90% (Kappa = 0.73).

### Feasibility and Safety

The test could be performed and interpreted both for WM and MPI in all enrolled patients, after the initial exclusion of 8 patients for poor acoustic windows or sulfonamide allergy. Feasibility of DASE was consequently 95%.

After dipyridamole infusion mild flushing or headache were commonly reported. There were no serious or irreversible adverse events. There was no suggestion for any direct adverse effect related to contrast infusion.

## Discussion

### Potential clinical value of detecting intermediate stenosis

An intermediate stenosis has a negative prognostic impact on future cardiac events only if associated with a reduced coronary (CFR) or fractional flow reserve (FFR); CFR or FFR have been found reduced in more than half of patients with known intermediate stenosis, non-invasively studied using stress-echocardiography (CFR assessment), or invasively studied with Doppler flow-wire measurements (FFR assessment) [2,4]. The detection of an intermediate stenosis may consequently be relevant for the clinical prognosis of patients, although the management of such intermediate stenosis needs to be tailored on a single patient basis.

The long-term prognostic value of MPI for hard cardiac events, independently by WM, has been clearly demonstrated [12,13], however no single study specifically addressed the prognostic value of a MPI defect subtended by an intermediate stenosis.

In conclusion, whether a reversible perfusion defect in patients with isolated intermediate stenosis implies an unfavorable prognosis is a question that remains unanswered.

### Wall motion criteria in intermediate stenosis

Contemporary studies using either dipyridamole or dobutamine echocardiography clearly demonstrated the suboptimal sensitivity of pharmacologic stress protocols to detect intermediate stenosis when using standard WM criteria [1,2,14,15]. In a study using MPI in conjunction with dobutamine-atropine SE, pooled sensitivity of WM criteria for intermediate stenosis was only 30%, increasing to 67% when MPI was added [1].

A recent study, in which high dose dipyridamole was used, reported a 20% sensitivity for WM criteria to detect 50%-75% coronary stenosis in patients with isolated intermediate stenosis of the anterior descending coronary artery; transthoracic Doppler CFR in this study was very effective to overcome the limited sensitivity of WM, but in the clinical practice the undisputable value of non-invasive CFR measurement is technically limited to the anterior coronary circulation [2].

The incremental value of contrast MPI is instead maintained for all coronary territories, as previously reported [1]. In our study the anterior circulation was affected in only 60% of patients with isolated intermediate stenosis.

Another study demonstrated a major step-up in sensitivity for detection of an intermediate stenosis (associated with fractional flow reserve < 0.75) by the use of contrast for left ventricle opacification during dobutamine echocardiography; in this case sensitivity increased from 48% for standard echocardiography without contrast, to 83% with the use of contrast for opacification, reflecting the usefulness of better endocardial border delineation [15].

Sensitivity data in this study, which is otherwise particularly interesting for the choice of a hemodynamic functional endpoint, are presumably much higher than real because of the bias of performing and interpreting the tests in patients with previously known intermediate stenosis, which was the reason for stress-testing.

### Clinical value of contrast MPI, costs and safety issues

Contrast use to date is not approved specifically for MPI by the European Medicines Agency (EMEA) and its use remains consequently investigational. In our study contrast MPI is useful to increase the sensitivity of pharmacologic stress-echocardiography when the diagnosis of less severe CAD/stenosis is the diagnostic endpoint, while for more severe CAD/stenosis the profound loss in specificity leads to a significant loss in accuracy, compared with standard WM analysis; whether the diagnostic cost-benefit profile of adding contrast MPI is worth the potential safety issues and contrast-related costs is still a matter of discussion. An isolated MPI abnormality (ie, with normal WM) should not be used as the sole indication to coronary angiography, due to its low specificity for epicardial obstructive CAD.

### False positives and coronary angiography

Patients with a positive provocative test (whatever stress-test considered) and no obstructive epicardial CAD at coronary angiography are usually defined as false-positives. It is now widely recognized that in many different clinical situations (microvascular disease, diffused coronary atherosclerosis, subcritical disease with coronary remodeling, myocardial bridges, coronary spasm, cardiomyopathies and others) coronary blood flow under stress condition may be significantly reduced in the absence of epicardial > 50% stenosis, up to the level capable to cause chest pain, ECG and perfusion abnormalities and, less frequently, WM abnormalities [16]. MPI is intrinsically incline to a higher "false positivity" rate than WM, due to its earlier role in the pathophysiology of the ischemic cascade.

Few stress-echocardiography studies reported that patients with so called "normal coronaries" and an abnormal stress-test (for WM or CFR-LAD) have a worse cardiac prognosis than patients with a normal test [17,18].

### Limitations

The specificity of WM in our study was lower than reported by several previous studies; this may be due to the non-conservative reading criteria adopted (≥1 segment with new dyssynergy in a region with normal rest function or worsening of rest dyssynergy), that were anyway similarly applied to MPI (≥1 segment with reversible MPI defect). Analysis of echocardiographic data was performed on a patient-basis and not on a territory-basis, since we felt it inappropriate, stress-echocardiograms being always interrupted in our centre at the very first sign of positivity, for safety reasons. The limitations of angiographic comparisons in relation to the discrepancy between stenosis severity and functional significance are pertinent to our study too. Use of angiographic data alone ignores patients with non-ischemic cardiomyopathies, the previous occurrence of coronary events in patients without significant stenosis or microcirculation disease. Wall motion abnormalities or MPI defects in these patients are classified as false-positives; in our experience this has a major impact on MPI data, more than on WM data.

## Conclusion

The current study shows that most of the incremental diagnostic sensitivity of MPI over WM analysis during DASE originates from patients with coronary artery stenosis of intermediate severity, defined as 50%-70% in luminal diameter. Interestingly, MPI maintains incremental sensitivity in the SEVERE group also, but clinically less significant. In the SEVERE group, however, the addition of MPI decreased the specificity more than how it increased the sensitivity, resulting in lower total accuracy, compared with WM assessment. The opposite is true when targeting coronary artery stenosis ≥50%.

We conclude that MPI should be used with caution if the endpoint of the test is the identification of angiographically severe (> 70%, so called "critical") stenosis. In this case the trade-off of MPI between sensitivity and specificity becomes unfavorable for MPI, while standalone WM assessment (using left ventricle contrast opacification, as we did in our study) maintains a higher total accuracy.

## Abbreviations

WM: wall motion; CAD: coronary artery disease; MPI: contrast myocardial perfusion imaging; DASE: dipyridamole-atropine stress echocardiography; QCA: quantitative coronary angiography; LVEF: left ventricular ejection fraction; CFR: coronary flow reserve; FFR: fractional flow reserve; RPP: rate-pressure product

## Competing interests

The authors declare that they have no competing interests.

## Authors' contributions

NG performed the echostress and drafted the manuscript, FR analyzed the CFR data, AS drafted the manuscript and performed the statistical analysis, FU made QCA analysis, CR drafted the manuscript. All authors read and approved the final manuscript

## Supplementary Material

Additional file 1 Abnormal MPI after dipyridamole with normal peak WM in a patient finally diagnosed with two-vessel 50%-70% coronary artery stenosis. Click here for file
